# Carotid Sinus Massage in Syncope Evaluation: A Nonspecific and Dubious Diagnostic Method

**DOI:** 10.5935/abc.20180134

**Published:** 2018-07

**Authors:** José Carlos Pachón Mateos

**Affiliations:** Universidade de São Paulo (USP), São Paulo, SP - Brazil; Hospital do Coração de São Paulo (HCor), São Paulo, SP - Brazil; Instituto Dante Pazzanese de Cardiologia, São Paulo, SP – Brazil

**Keywords:** Syncope/etiology, Syncope/physiopathology, Carotid sinus/physiology, Arrhythmias, Cardiac/complications

The study by Wu et al.^1^ questions the value of carotid sinus massage (CSM) for the investigation of syncope. It was well conducted, with two reasonably equivalent groups, with and without previous syncope, submitted to the same type of bilateral CSM, under rigorous evaluation of symptoms, cardiac rhythm and blood pressure. The authors found no difference in the response to CSM between the two groups. They concluded that CSM in the assessment of unexplained syncope would be a nonspecific and dubious diagnostic method. The results are clear and well structured. We agree with the authors’ conclusion about the study findings. The limitations suggested are rightful. There is no doubt that CSM is an empiric method, of uncertain results, and this type of study serves to alert to its drawbacks. However, why is CSM still included in the guidelines? Certainly because it is a simple, well-tolerated, low-cost, low-risk procedure as long as the technique and the contraindications are respected, and can be performed rapidly during tilt-test, establishing the diagnosis in up to 30% of elderly patients with syncope of unknown origin.^2^ However, as any other investigative method, it has important limitations that should be addressed cautiously. It is worth noting that the response to CSM depends on several investigator’s and patient’s factors, having value only when positive, when reproducing the spontaneous symptoms and when the patient’s clinical findings are compatible with reflex syncope. In addition, it has no power of exclusion.

Thus, despite these drawbacks, CSM continues valid according to the European Society of Cardiology guidelines, which consider it is indicated as class I, level of evidence B, for patients older than 40 years of age with syncope of unknown origin compatible with reflex origin.^3^ The diagnosis of carotid sinus syndrome (CSS) is confirmed if the CSM causes bradycardia (asystole) and/or hypotension that reproduce the spontaneous symptoms, and if the patients have clinical features compatible with the reflex mechanism of syncope, class I, level of evidence B. Although neurological complications are rare, the maneuver should be avoided in patients who already had an ischemic stroke, have a carotid murmur or important carotid vasculopathy. The history of syncope with positive CSM reproducing the symptoms confirms the diagnosis of CSS. However, a positive CSM without a history of syncope characterizes carotid sinus hypersensitivity, which, in elderly patients with unexplained syncope, can be a nonspecific finding and should be considered cautiously in the assessment of the syncope mechanism, because it is present in up to 40% of the cases.^4^

The American College of Cardiology/American Heart Association/Heart Rhythm Society guideline for the assessment and treatment of patients with syncope also considers CSM necessary for the diagnosis of CSS,^5^ which is established by the reproduction of syncope during the maneuver in the presence of a cardioinhibitory response > 3 seconds, of atrioventricular block, of a significant vasodepressor response (a reduction ≥ 50 mmHg in systolic pressure) or of the association with mixed response.

It is worth noting that, in our clinical practice, we have observed that the vasodepressor response measured by the absolute decline in systolic pressure to ≤ 85 mmHg seems more specific than the relative decline of 50 mmHg traditionally considered in several studies. This was reported by the authors in the present article.

Thus, we consider that the present study has great value to draw the attention of the specialist to the limitations of the CSM.
